# Grafting cucumber onto luffa improves drought tolerance by increasing ABA biosynthesis and sensitivity

**DOI:** 10.1038/srep20212

**Published:** 2016-02-02

**Authors:** Shanshan Liu, Hao Li, Xiangzhang Lv, Golam Jalal Ahammed, Xiaojian Xia, Jie Zhou, Kai Shi, Tadao Asami, Jingquan Yu, Yanhong Zhou

**Affiliations:** 1 Department of Horticulture, Zijingang Campus, Zhejiang University, 866 Yuhangtang Road, Hangzhou, 310058, P.R. China; 2 College of Horticulture, Northwest A&F University, Yangling 712100, P.R. China; 3 Department of Applied Biological Chemistry, University of Tokyo, Bunkyo Ku, Tokyo 1138657, Japan; 4 Zhejiang Provincial Key Laboratory of Horticultural Plant Integrative Biology, 866 Yuhangtang Road, Hangzhou, 310058, P.R. China

## Abstract

Balancing stomata-dependent CO_2_ assimilation and transpiration is a key challenge for increasing crop productivity and water use efficiency under drought stress for sustainable crop production worldwide. Here, we show that cucumber and luffa plants with luffa as rootstock have intrinsically increased water use efficiency, decreased transpiration rate and less affected CO_2_ assimilation capacity following drought stress over those with cucumber as rootstock. Drought accelerated abscisic acid (ABA) accumulation in roots, xylem sap and leaves, and induced the transcript of ABA signaling genes, leading to a decreased stomatal aperture and transpiration in the plants grafted onto luffa roots as compared to plants grafted onto cucumber roots. Furthermore, stomatal movement in the plants grafted onto luffa roots had an increased sensitivity to ABA. Inhibition of ABA biosynthesis in luffa roots decreased the drought tolerance in cucumber and luffa plants. Our study demonstrates that the roots of luffa have developed an enhanced ability to sense the changes in root-zone moisture and could eventually deliver modest level of ABA from roots to shoots that enhances water use efficiency under drought stress. Such a mechanism could be greatly exploited to benefit the agricultural production especially in arid and semi-arid areas.

Drought is one of the major limitations to agricultural production worldwide, especially in arid and semi-arid areas. Additionally, uneven distribution of rainfall due to global climate change and injudicious utilization of water resource by increasing human population have aggravated the frequency and severity of drought in many areas^1^,^2^. To secure a desirable harvest under drought, approaches for improving plant water use efficiency (WUE) are of great significance in sustainable agriculture.

Stomata play a major role in regulating CO_2_ uptake and transpirational water loss in plants. Genetic manipulation of stomatal development and movement has been shown to alter WUE by regulating transpiration which eventually affects drought tolerance^3^,^4^,^5^. Stomatal movement is regulated by many environmental factors as well as phytohormones^6^,^7^. Among them, abscisic acid (ABA) is well recognized to mediate early response to soil drying by limiting stomatal aperture to prevent water loss^8^,^9^,^10^. In recent years, a significant progress has been made in understanding the molecular mechanisms of ABA perception and signal transduction in *Arabidopsis thaliana*^11^. Initially, ABA binds with PYR1/PYL/RCAR (PYRABACTIN RESISTANCE1/PYR1-LIKE/REGULATORY COMPONENTS OF ABA RECEPTORS) to promote interaction with PP2C (type 2C protein phosphatase), thereby inhibiting the phosphatase activity of PP2C; as a result, phosphorylated SnRK2s (Sucrose nonfermenting1-related protein kinase2) can activate the relevant transcription factors^12^. ABA-induced rapid stomatal responses are associated with the production of reactive oxygen species (ROS) and are controlled by the protein kinases OPEN STOMATA 1 (OST1) and SLOW ANION CHANNEL-ASSOCIATED 1 (SLAC1)^13^.

Several studies demonstrated that an increase in endogenous ABA accumulation by overexpression of 9-cis-epoxycarotenoid dioxygenase (*NCED*) genes involved in ABA biosynthesis reduced transpiration and increased WUE in various plant species^14^,^15^,^16^,^17^. However, those transgenic plants showed reduced CO_2_ assimilation rate on which crop productivity largely depends. Therefore, genetic manipulation to balance photosynthesis and WUE offers much complexity under drought stress.

Recent studies have firmly established the role of the plant vascular system as an effective communication channel that transmits both root- and shoot-borne long-distance signals such as hormones under various abiotic and biotic conditions^18^. Such signals from roots can warn aboveground tissues about the existing below ground environment and vice-versa. Previous studies on grafting revealed that scion transpiration rate and its acclimation to water deficit are controlled genetically by the rootstock, through different genetic architectures in grapevine^19^. ABA has been suggested to play a critical role in the root-shoot communication in responses to water deficit^18^,^19^,^20^. In citrus, the tetraploid clones show increased tolerance to drought over diploid clones, which is evidenced by modified expression of the genes in the roots that regulate long-distance ABA signaling and adaptation to stress^20^. There are evidences that shoot transpiration rate is largely dependent on the delivery of ABA from the roots and the sensitivity to ABA in response to water deficit^8^. Therefore, selection of genotypes with appropriate ABA biosynthesis and sensitivity as well as enhanced drought tolerance remains as a challenge to the plant breeders^21^. In this regard, grafting drought-sensitive genotype onto rootstock with increased ABA synthesis capacity or sensitivity in response to drought could be a useful approach to improve WUE and crop productivity.

Cucumber (*Cucumis sativus* L.) is an important vegetable crop worldwide. Being originated from Himalayas range basin, cucumber plants show high transpiration rate and sensitivity to drought. Several studies showed that grafting cucumber onto figleaf gourd and luffa could improve tolerance of cucumber plants against chilling or high temperature; however, relatively little is known how water utilization efficiency is influenced by rootstock^22^,^23^,^24^. Our preliminary experiments and field trail revealed that cucumber plants grafted onto luffa (*Luffa cylindrica* Roem.), which is widely cultivated in the areas with drought and insufficient rainfall, showed enhanced tolerance against drought compared with figleaf gourd, pumpkin and cucumber as rootstocks (unpublished). In this study, we compared the response of reciprocally grafted cucumber and luffa plants to drought stress through investigation of photosynthesis, transpiration, WUE and ABA signaling cascades. The mechanism of ABA-mediated drought tolerance in luffa rootstock- grafted cucumber was also discussed.

## Results

### Grafting onto luffa rootstock increases tolerance against drought

**Figure 1:**
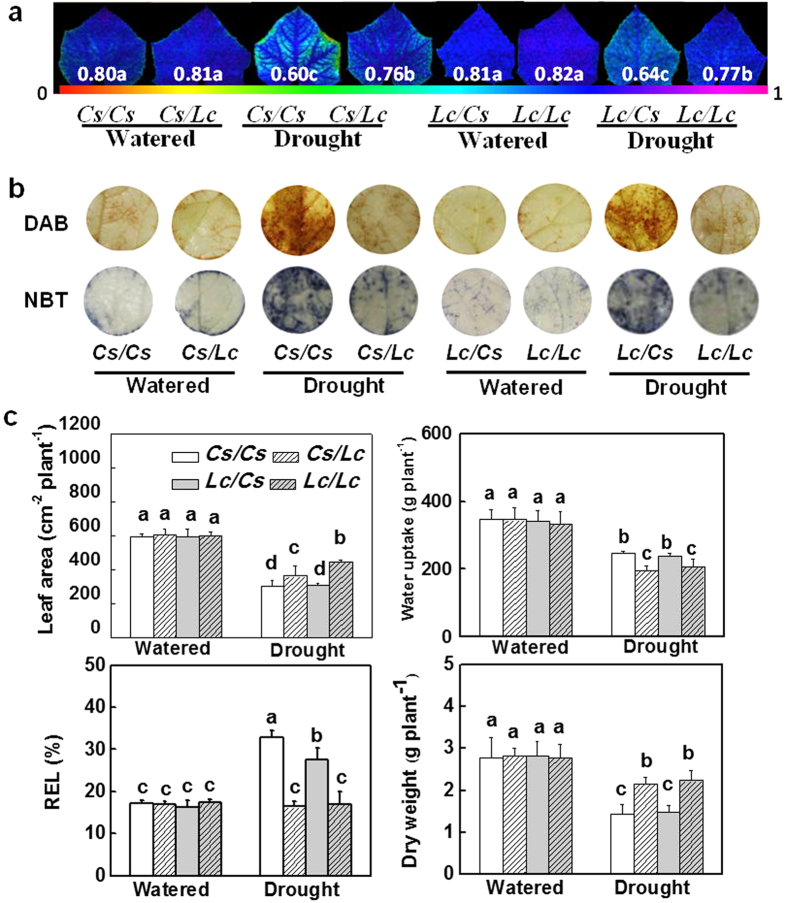
Responses of cucumber and luffa rootstocks to drought stress. (**a**) The maximum photochemical efficiency of PSII (Fv/Fm). The underneath false colour code depicted in the image, ranges from 0 (black) to 1 (purple). (**b**) Accumulation of H_2_O_2_ [3,3′-diaminobenzidine (DAB) staining, upper panel] and O_2_^−^ [nitroblue tetrazolium (NBT) staining, lower panel]. (**c**) The relative electrolyte leakage (REL), water uptake and leaf area per plant, and dry weight. Grafted plants were well watered or drought stressed by withholding water for 9 d. All data were determined 9 d after drought treatment and presented as the mean of four biological replicates (±SD). Different letters indicate significant differences (*P* < 0.05) according to the Tukey’s test.

To examine whether plant tolerance to drought is altered by rootstock or scion, we performed reciprocal grafting between cucumber (*Cs*) and luffa (*Lc*) seedlings. Grafted seedlings with similar leaf area per plant were irrigated to 50% substrate water content (SWC) and then allowed to grow without further water supply for 9 d. As shown in Fig. 1, there were no significant differences in the maximum photochemical efficiency of photosystem II (PSII) (Fv/Fm), accumulation of superoxide (O_2_^−^) and hydrogen peroxide (H_2_O_2_), relative electron leakage (REL) and plant biomass in *Cs/Cs* and *Cs/Lc* plants as well as in *Lc/Lc* and *Lc/Cs* plants under well-irrigated condition. However, in response to water deficit, *Cs/Lc* plants and *Lc/Lc* plants showed delayed leaf wilting, decreased accumulation of O_2_^−^ and H_2_O_2_ and water uptake per plant, and increased Fv/Fm, leaf area and dry matter accumulation as compared to *Cs/Cs* plants and *Lc/Cs* plants, respectively (Fig. 1 and Supplementary Fig. S1). For example, after water withholding for 9 d, all *Cs/Cs* plants wilted with remarkable reductions in dry matter accumulation and Fv/Fm that accounted for 24.4% and 76.9%, respectively (Fig. 1 and Supplementary Fig. S1). Unlike *Cs/Cs* plants, *Cs/Lc* plants did not wilt and its reductions in dry matter accumulation and Fv/Fm were negligible (5.8 and 3.8%, respectively). Furthermore, *Cs/Lc* and *Lc/Lc* plants showed increased activities of superoxide dismutase (SOD), dehydroascorbate reductase (DHAR) and guaiacol peroxidase (GPOD) compared with *Cs/Cs* and *Lc/Cs* plants in the leaves, respectively (Supplementary Fig. S2). These results imply that plant tolerance to drought is mainly dependent on rookstock and *Lc* rootstock has the potential to enhance drought tolerance.

**Figure 2:**
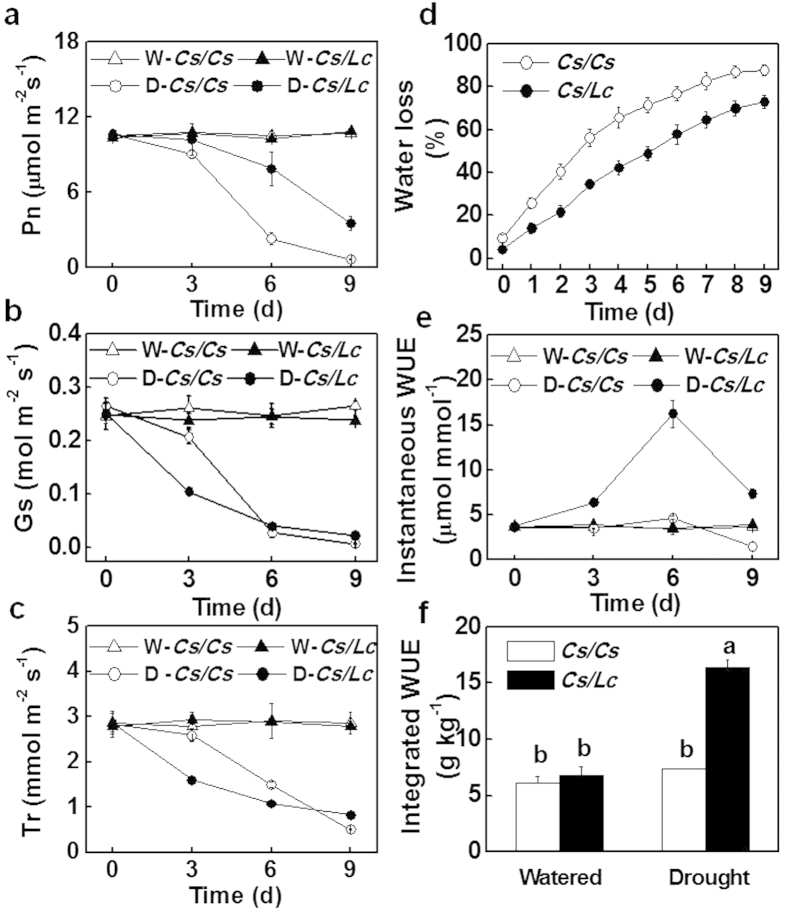
Changes in photosynthesis and water use efficiency of cucumber plants as influenced by rootstock. CO_2_ assimilation rate (Pn, **a**), stomatal conductance (Gs, **b**), transpiration rate (Tr, **c**), water loss in growth substrate (**d**), instantaneous WUE (**e**) and integrated WUE (**f**) of cucumber grafted plants under well watered and drought stress conditions. Intact plants were well watered (W-*Cs/Cs* and W-*Cs/Lc*) or drought stressed by withholding water (D-*Cs/Cs* and D-*Cs/Lc*) for 9 d. Integrated WUE were determined 9 d after water withholding. Data are presented as the mean of four biological replicates (±SD). Different letters indicate significant differences (*P* < 0.05) according to the Tukey’s test.

*Cs/Lc* plants exhibited higher CO_2_ assimilation rate (Pn) but lower stomatal conductance (Gs) and transpiration rate (Tr) as compared to *Cs/Cs* plants in response to water deficit (Fig. 2a–c). At 9 d after water with holding, Pn for *Cs/Cs* and *Cs/Lc* plants was 93.7 and 67.4% lower than those in the well watered plants, respectively. A detailed analysis in the loss of water in growth substrate revealed that *Cs/Cs* plants absorbed more water than *Cs/Lc* plants and this was associated with an increased transpiration rate as compared to *Cs/Lc* plants (Fig. 2d). An estimation of WUE based on CO_2_ assimilation rate and transpiration rate, i.e. instantaneous WUE, revealed that *Cs/Lc* plants had a higher leaf WUE than *Cs/Cs* plants throughout the experiment (Fig. 2e,f). Moreover, integrated WUE calculated from the dry matter gain and water loss from the substrate was also higher (ca. 122.4%) in *Cs/Lc* plants than that in *Cs/Cs* plants after withholding water for 9 d. Thus, water deficit resulted in a more significant increase in WUE for *Cs/Lc* plants as compared to *Cs/Cs* plants and such increase in WUE for *Cs/Lc* plants was not in expense of photosynthesis. Similarly, higher Pn and WUE with lower Gs, Tr and water loss were observed in *Lc/Lc* plants as compared to *Lc/Cs* plants (Supplementary Fig. S3). These results suggest that *Cs* roots had poor ability to adapt the changes in soil drought while *Lc* rootstock could enhance drought tolerance and water use efficiency in response to water deficit by decreasing transpiration.

### Changes in stomatal movement and stomatal development in response to drought

**Figure 3:**
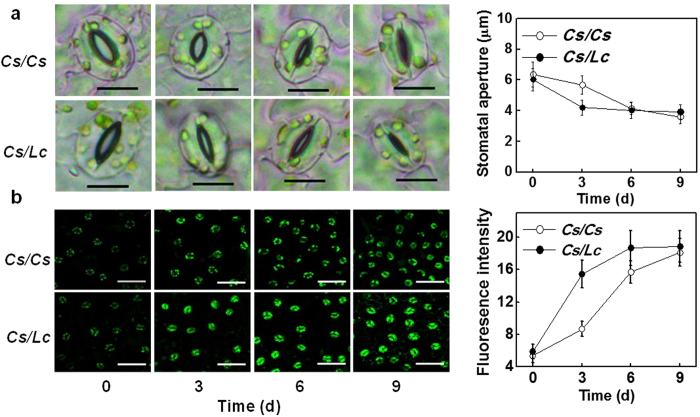
Stomatal aperture (a) and H_2_O_2_ accumulation (b) in cucumber leaves as influenced by rootstock under drought stress. Scale bar in (**a**) = 15 μm. Scale bar in (**b**) = 50 μm. Intact plants were drought stressed by withholding water for 9 d. Data are presented as the mean of four biological replicates (±SD). Different letters indicate significant differences (*P* < 0.05) according to the Tukey’s test.

We then investigated how the changes in transpiration were associated with the stomatal movement and stomatal development in the grafted plants. Under well watered conditions, *Cs/Lc* plants showed no differences in stomatal aperture with *Cs/Cs* plants (Fig. 3a). Drought induced marked decreases in the stomatal aperture for both *Cs/Cs* and *Cs/Lc* plants. Although water-break-induced stomatal closure was more significant at 3 d, no significant differences were found at 6 and 9 d, and stomatal aperture was significantly lower in *Cs/Lc* plants than that in *Cs/Cs* plants at 3 d. Similarly, stomatal aperture was also significantly lower in luffa plants grafted onto its own roots in comparison with *Cs* rootstock after exposure to water deficit (Supplementary Fig. S4a). In addition, the water deficit treatment decreased the stomatal density in new leaves of *Cs/Lc* plants, but not in those of *Cs/Cs* plants (Supplementary Fig. S5a,b).

We also tried to unveil possible relationship between drought-induced stomatal movement and H_2_O_2_ accumulation in the stomata. During initial stage of water withholding, H_2_O_2_ accumulation in the stomata of *Cs/Lc* plants was not different from that of *Cs/Cs* plants (Fig. 3b). With the progression of drought, however, stomatal H_2_O_2_ accumulation in both *Cs/Cs* and *Cs/Lc* leaves increased gradually, with prominent increases in *Cs/Lc* plants. No obvious difference in stomatal aperture and H_2_O_2_ accumulation were noticed between *Cs/Cs* and *Cs/Lc* plant at the advanced stage of drought (6 d and 9 d). Similarly, *Lc/Lc* plants had higher stomatal H_2_O_2_ level than *Lc/Cs* plants in response to water deficit (Supplementary Fig. S4b). It is quite possible that increased accumulation of H_2_O_2_ in stomata which was obvious at 3 d in *Lc/Cs* plants might have a role in rapid stomatal closure.

### ABA accumulation in leaves, roots and xylem saps in response to drought

ABA is an important chemical signal involved in response to drought in plants. We analysed the changes in ABA accumulation in roots, xylem saps and leaves as well as the transcript levels of *9-cis- epoxycarotenoid dioxygenase2* (*NCED2*), a key rate-limiting gene for ABA biosynthesis, in the leaves of plants after exposure to water deficit.

**Figure 4:**
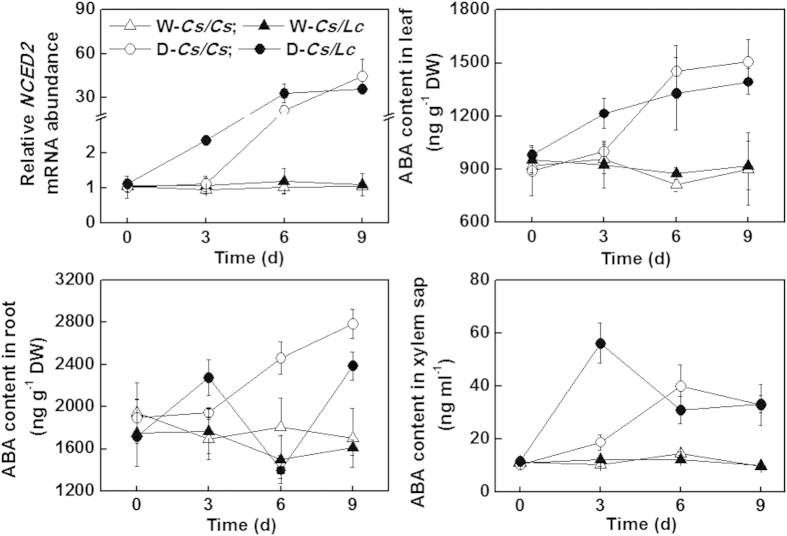
Transcript abundance of ABA biosynthetic gene in leaves and the content of ABA in leaves, roots and xylem sap as influenced by rootstock under drought stress. Intact plants were well watered (W-*Cs/Cs* and W-*Cs/Lc*) or drought stressed (D-*Cs/Cs* and D-*Cs/Lc*) by withholding water for 9 d. Data are presented as the mean of four biological replicates (±SD). Different letters indicate significant differences (*P* < 0.05) according to the Tukey’s test.

Under well watered conditions, rootstock genotypes did not alter the accumulation of ABA in the leaves, roots and xylem sap. In response to water deficit, all the grafted plants showed an increase in ABA accumulation in the leaves, roots and xylem saps (Fig. 4 and Supplementary Fig. S6). Significantly, the increase in ABA accumulation in the leaves, roots and xylem saps was found as early as at 3 d after exposure to water deficit and no further increase in leaf ABA contents occurred with the progression of drought stress in *Cs/Lc* and *Lc/Lc* plants. In comparison, a continuous increase in ABA accumulation in the leaves, roots and xylem saps was found in *Cs/Cs* and *Lc/Cs* plants from day 6 after exposure to water deficit. Finally, *Cs/Cs* and *Lc/Cs* plants had higher ABA accumulation both in the roots and leaves than that in *Cs/Lc* and *Lc/Lc* plants, respectively, whilst ABA concentration in the xylem sap neither differed between the *Cs/Cs* and *Cs/Lc* plants nor between *Lc/Cs* and *Lc/Lc* plants. Meanwhile, transcript of *NCED2* increased gradually with the progression of water deficit both in *Cs/Cs* and *Cs/Lc* plants. Like ABA accumulation, water deficit-induced increase in *NCED2* transcript occurred later in *Cs/Cs* plants than in *Cs/Lc* plants. Surprisingly, the new leaves in *Cs/Lc* plants had an increased ABA contents over *Cs/Cs* plants after exposure to water deficit conditions (Supplementary Fig. S5c).

### Inhibition of ABA biosynthesis compromises luffa rootstock-induced drought tolerance

**Figure 5:**
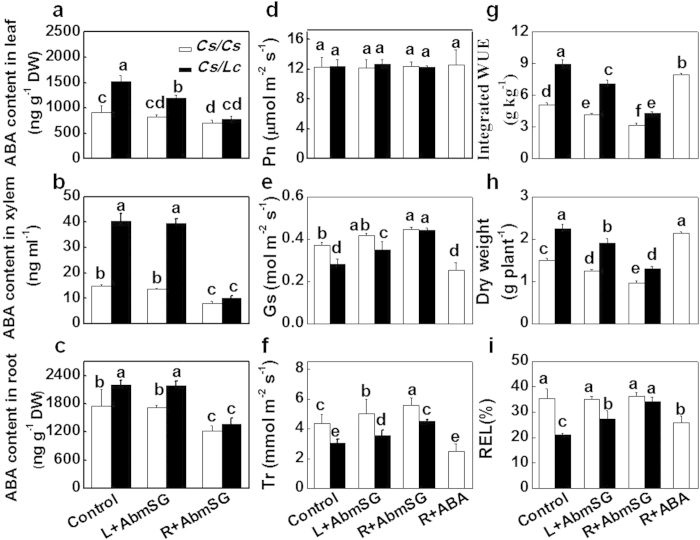
Changes in ABA accumulation, drought tolerance and plant water use efficiency as influenced by foliar application (L) or root application (R) of ABA biosynthesis inhibitor AbamineSG (AbmSG)) or ABA. ABA accumulation in leaves (**a**), ABA accumulation in xylem sap (**b**), ABA accumulation in roots (**c**), CO_2_ assimilation rate (Pn, **d**), stomatal conductance (Gs, **e**), transpiration rate (Tr, **f**), integrated WUE (**g**), dry matter (**h**), and relative electrolyte leakage (REL, **i**) in cucumber grafted plants after water withholding. ABA accumulation and leaf gas exchange were determined at 3 d after water withholding. Integrated WUE, dry matter accumulation and relative electrolyte leakage were determined at 9 d after water withholding. At the 2^nd^ and 5^th^ d after the initiation of the drought treatment, shoots were sprayed with 50 μM AbamineSG (5 ml per plant), whereas roots of others were irrigated with 5 μM AbamineSG (50 ml per plant) or 10 μM ABA (50 ml per plant). An equal amount of water was applied as the control. Data are presented as the mean of four biological replicates (±SD). Different letters indicate significant differences (*P* < 0.05) according to the Tukey’s test.

To determine whether the increased biosynthesis of ABA in the luffa rootstock plays a role in the drought tolerance, we determined the changes in drought tolerance in *Cs/Cs* and *Cs/Lc* plants with irrigation or foliar application of abamineSG, an inhibitor of ABA biosynthesis^25^. Under well-watered conditions, irrigation with or foliar application of abamineSG did not affect the growth and water uptake in both grafting combinations (Supplementary Fig. S7). Under drought stress conditions, foliar application of AbamineSG did not alter ABA accumulation in roots and the ABA contents in the xylem saps both in *Cs/Cs* and *Cs/Lc* plants (Fig. 5b,c). Root application with AbamineSG decreased ABA accumulation in roots, leaves and the xylem saps both in *Cs/Cs* and *Cs/Lc* plants (Fig. 5a–c). Both foliar and root application of abamineSG did not alter Pn but differentially increased Gs and Tr. The effects were more significant in *Cs/Lc* plants with root application of abamineSG at 5 d after exposure to water deficit treatment (Fig. 5d–f). Finally, *Lc* rootstock-induced increases in dry matter accumulation, leaf area and WUE, and the decrease in relative electron leakage (REL) were differentially compromised by foliar and root application of AbamineSG (Fig. 5g–i; Supplementary Fig. S7b). Notably, these effects were more significant in *Cs/Lc* plants and in plants with root application of abamineSG. However, under drought conditions, irrigation with or foliar application of abamineSG did not affect water uptake of plant in both grafting combinations with the exception for *Cs/Lc* plants irrigated with abamineSG (Supplementary Fig. S7c). Meanwhile, root application of ABA significantly improved plant growth and drought tolerance, which was associated with decreased Tr, Gs and REL and increased dry matter accumulation and WUE (Fig. 5 and Supplementary Fig. S7).

### Increased ABA sensitivity in *Cs/Lc* plants

**Figure 6:**
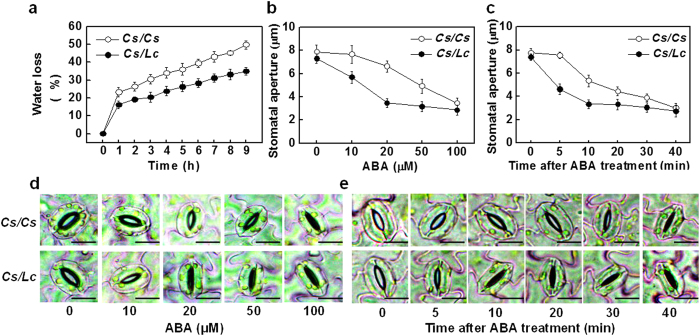
The leaf water loss and sensitivity of stomatal aperture to ABA as influenced by rootstock. (**a**) Water loss during a 9-h period from the detached leaves; water loss is expressed as the percentage of initial fresh weight of detached leaves. (**b,d**) The effect of ABA concentration on stomatal aperture in detached abaxial epidermal strips. Scale bar = 25 μm. (**c,e**) Time-course changes in stomatal aperture in detached abaxial epidermal strips in response to 50 μM ABA. Scale bar = 25 μm. Data are presented as the mean of four biological replicates (±SD). Different letters indicate significant differences (*P* < 0.05) according to the Tukey’s test.

To test whether rootstock-induced stomatal movement was related to their sensitivity to ABA, we then compared the water loss of detached leaves from *Cs/Cs* and *Cs/Lc* plants. Time-course analysis showed that leaves from *Cs/Cs* plant had a more significant water loss over time than those from *Cs/Lc* plants (Fig. 6a). Meanwhile, stomatal closure could be induced by exogenous ABA at a concentration as low as 10 μM in *Cs/Lc* plants whilst that for *Cs/Cs* plants was 50 μM (Fig. 6b,d). Time-course quantification also revealed that stomata in the leaves of *Cs/Lc* closed within 5 min in response to 50 μM ABA while it required more than 5 min to induce stomatal closure in *Cs/Cs* leaves (Fig. 6c,e). These results suggest that stomata in *Cs/Lc* plants had an increased sensitivity to ABA over that in *Cs/Cs* plants.

### Response of drought tolerance index and ABA signaling cascade to various levels of water deficit

**Figure 7:**
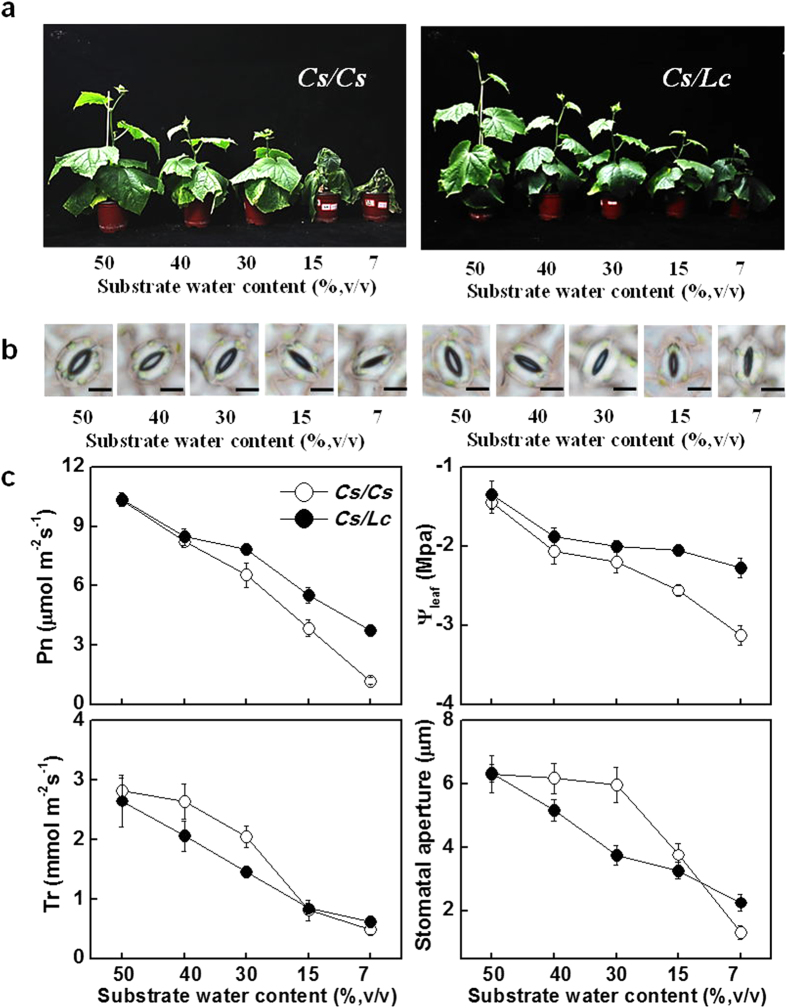
Effect of rootstock on the tolerance to drought stress with different growth substrate water content in cucumber. (**a**) Phenotypes of cucumber plants with its own roots (*Cs/Cs*) and luffa roots (*Cs/Lc*) as rootstocks under different substrate water content conditions. (**b**) Stomatal aperture as influenced by rootstock and substrate water content. Scale bar = 15 μm. (**c**) CO_2_ assimilation rate (Pn), transpiration rate (Tr), leaf water potential (ψ) and stomatal aperture as influenced by rootstock and substrate water content. All data were determined 7 d after drought treatment and are presented as the mean of four biological replicates (±SD). Different letters indicate significant differences (*P* < 0.05) according to the Tukey’s test.

To elucidate a comprehensive response of *Lc* rootstock to specific level of water deficit, we exposed the grafted plants to different substrate water content (SWC). As shown in Fig. 7a, plant growth was suppressed with the decrease in SWC for both *Cs/Cs* and *Cs/Lc* plants. However, *Cs/Lc* plants showed increased tolerance to water deficit as noticed by the light wilting, higher Pn and leaf water potential (ψ_leaf_) at 30, 15 and 7% SWC (Fig. 7a,c). Meanwhile, *Cs/Lc* plants exhibited decreased Tr, Gs and stomatal aperture over *Cs/Cs* at 40 and 30% SWC (Fig. 7b,c and Supplementary Fig. S8). Furthermore, water deficit induced an increase in leaf temperature for both *Cs/Cs* plants and *Cs/Lc* plants. However, the leaf temperature of *Cs/Lc* plants was significantly higher than those for *Cs/Cs* plants at 40 and 30% SWC, suggesting a higher degree of transpiration reduction in *Cs/Lc* plants (Supplementary Fig. S8). All these results again suggested that the stomata of *Cs/Lc* plants were more sensitive to the changes in water content of the growth substrate than those of *Cs/Cs* plants and grafting onto luffa roots could improve drought tolerance which was associated with stomatal movement-driven reduction in transpiration in *Cs/Lc* plants.

**Figure 8:**
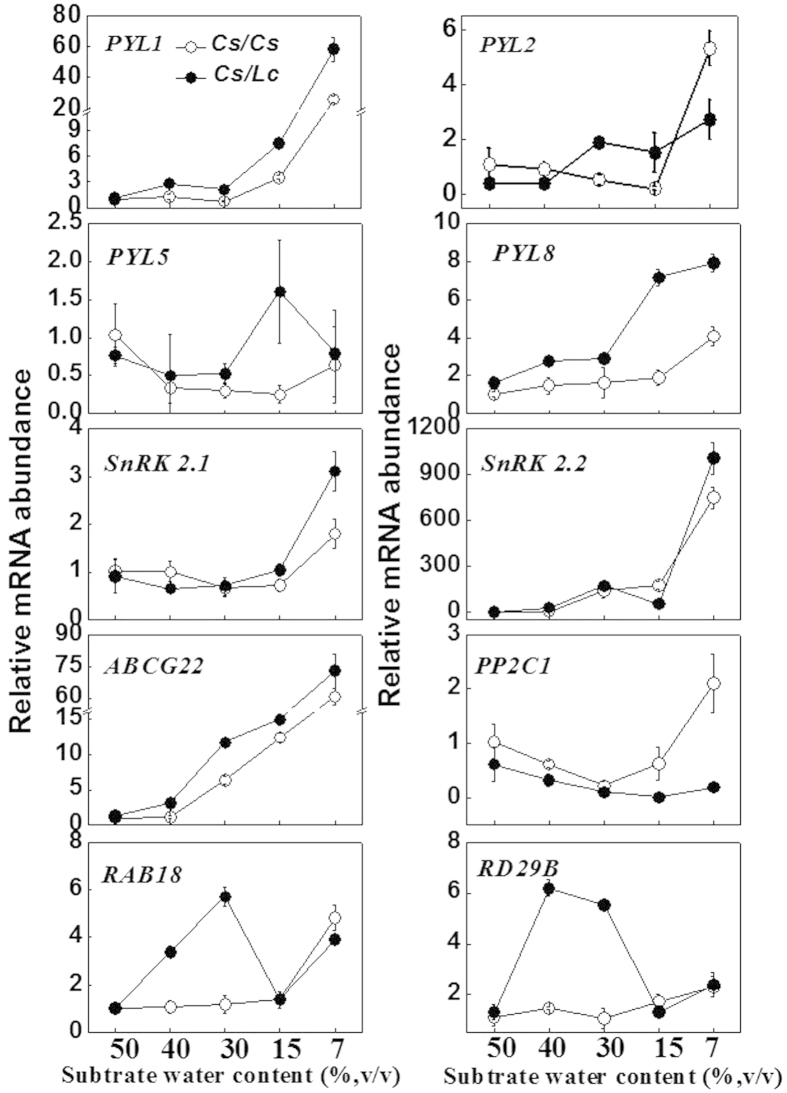
Transcript abundance of ABA signaling genes as influenced by rootstock under different levels of water deficit. All transcripts were determined 7 d after drought treatment. Data are presented as the mean of four biological replicates (±SD). Different letters indicate significant differences (*P* < 0.05) according to the Tukey’s test.

To further determine rootstock-induced changes in the ABA signaling in response to different SWC in grafted plants, transcripts of 10 genes involved in ABA- and/or drought stress-response were analyzed after exposure of grafted cucumber plants to water deficit (Fig. 8). Transcripts of most of these genes increased with the decrease in SWC both in *Cs/Cs* and *Cs/Lc* plants, while the increases being more significant in *Cs/Lc* plants. Among them, transcripts of ABA receptors genes (*PYL1, PYL2, PYL5* and *PYL8*)^26^ and ABA transporter gene (*ABCG22*)^26^ were profusely induced in *Cs/Lc* plants compared with those in *Cs/Cs* plants throughout the water deficit treatment. *SnRK 2.1* and *SnRK 2.2*, two ABA signaling-related genes^26^, were largely induced in *Cs/Lc* plants under 7% SWC condition as compared to *Cs/Cs* plants. Transcripts of the typical ABA-dependent genes (*RAB18* and *RD29B*)^26^ in *Cs/Lc* plants were markedly higher than those in the *Cs/Cs* plants under mild water deficit conditions (40% and 30% SWC) but were not significantly different under severe water deficit conditions (15% and 7%). In contrast, transcript level for *PP2C1*, a negative regulator of ABA singaling^26^, decreased with the progression of water deficit with the exception for *Cs/Cs* plants under 7% SWC condition, and was much lower in *Cs/Lc* plants than in *Cs/Cs* plants. These findings demonstrate that luffa rootstock plays a positive role in drought stress adaptation in cucumber plants by modulating ABA-dependent signaling cascades.

## Discussion

Grafting onto resistant rootstocks could improve plant tolerance to a variety of abiotic stresses; however, the underlying mechanisms of such enhanced tolerance are largely unknown^18^,^19^,^27^,^28^. Here, we found that cucumber plants with luffa as rootstocks had an increased tolerance against drought as indicated by the increased biomass accumulation and decreased accumulation of ROS such as O_2_^−^ and H_2_O_2_ under drought stress (Figs 1 and 7). Previously, several studies demonstrated that grafting with drought tolerant rootstocks could improve tolerance against drought which is associated with increased ROS scavenging capacity in the shoots^27^,^28^. In our study, there was less accumulation of O_2_^−^ and H_2_O_2_ whilst activity of several antioxidant enzymes was higher in the leaves of *Cs/Lc* plants as compared to *Cs/Cs* plants under drought (Fig. 1b and Supplementary Fig. S2). The higher leaf water potential (ψ_leaf_) for *Cs/Lc* plants over *Cs/Cs* plants observed in our study suggested that *Cs/Lc* plants might face low water stress and thus accumulating low ROS in comparison with *Cs/Cs* plants (Figs 1b and 7c). Accordingly, the low accumulation of ROS in *Cs/Lc* plants over *Cs/Cs* plants could be attributed to the decreased ROS production and increased ROS scavenging. ABA is actively involved in the regulation of anti-oxidation reactions by modulating enzymatic and non-enzymatic antioxidants in plants^29^. The faster and greater induction of ABA accumulation and ABA signaling in *Cs/Lc* plants in response to water deficit suggested that ABA could partly contribute to the decreased ROS accumulation as well as increased activity of antioxidant enzymes. Nonetheless, increased accumulation of stomatal H_2_O_2_ in *Cs/Lc* plants during early stage of drought (Fig. 3), might indicate a signaling role of ROS that could be potentially induced by ABA for rapid stomatal closure^13^,^30^.

Till date, our knowledge on the mechanism of the grafting-induced drought tolerance is fragmentary. Improved drought tolerance could be attributable to improved ability for the roots to absorb water and decreased transpiration in the above ground plant parts especially leaves under drought^31^,^32^,^33^. In the current study, we found that *Cs/Lc* plants did not have an increased ability to absorb water as indicated by the low water loss from the growth substrate (Fig. 2a). Time-course revealed that *Cs/Lc* plants always showed decreased transpiration rate over *Cs/Cs* plants under drought conditions. Transpiration is largely controlled by stomatal movement and the stomatal development in the leaves^6^,^34^. Stomatal development is usually accomplished during early leaf ontogeny. In agreement with this, there was no significant difference in the stomatal density in the mature leaves (data not shown). However, the newly developed leaves in *Cs/Lc* plants had a decreased stomatal density over *Cs/Cs* plants, suggesting that the decreased transpiration at the later stage of experiment was partly attributed to the stomatal development in *Cs/Lc* plants (Supplementary Fig. S6). In Arabidopsis, several ABA deficient or signaling mutants showed increased stomatal density over the wild type plants^35^,^36^,^37^. It is plausible that drought-induced ABA played a critical role in the altered stomatal development in the grafted plants. Importantly, *Cs/Lc* plants had a lower stomatal conductance under drought conditions, and stomatal closure was induced earlier in *Cs/Lc* plants as compared to *Cs/Cs* plants in spite of the higher leaf water potential. Thus, *Cs/Lc* plants have developed an increased ability to sense drought stress over *Cs/Cs* plants.

Stomata are important not only for transpiration but also for photosynthesis which contributes greatly to the WUE in a plant^6^,^38^. A reduction in stomatal conductance is usually followed by a decrease in CO_2_ assimilation rate in the leaves, leading to decrease or increase in WUE, depending on the severity of stress and plant species^39^. In our study, *Cs/Lc* plants always showed lower stomatal conductance together with lower stomatal aperture but higher CO_2_ assimilation rate, leading to an increased instantaneous WUE over *Cs/Cs* plants under water deficit. The high WUE for *Cs/Lc* plants under water deficit was also observed in experiment with intact plants (i.e. Integrated WUE). These results indicate that a moderate decrease in stomatal aperture in the *Cs/Lc* plants under drought conditions can reduce the loss of water without a concomitant effect on the supply of CO_2_ for carbon assimilation, resulting in higher instantaneous and integrated WUE in plants (Fig. 2). This was consistent with the previous study that decreased stomatal conductance within a given range could reduce transpiration without altering net photosynthesis rate in Arabidopsis^5^,^34^.

It is well established that the plant hormone ABA plays essential role in plant responses to drought by influencing stomatal development and movement^40^,^41^. Therefore, any changes in ABA biosynthesis or signaling are expected to alter drought tolerance in plants. Under field conditions, drought tolerance could be modified by root architectures and grafting-induced drought tolerance is not necessarily associated with changes in water utilization efficiency^19^. On the other hand, ABA-induced stomatal movement is generally associated with alteration in WUE as shoot transpiration rate is largely dependent on the delivery of ABA from the roots and the sensitivity to ABA in response to water deficit^8^. In citrus, tetraploid clones show increased tolerance to drought over diploid citrus clones, which is attributed to the high ability of tetraploid clones to synthesize ABA in the roots^20^. In our study, a faster induction of ABA content was observed in the roots, leaves and xylem saps of *Cs/Lc* plants as compared to *Cs/Cs* plants in response to drought stress, which was consistent with rapid upregulation of ABA biosynthetic gene *NCED2* in leaves (Fig. 4). These observations suggest that luffa rootstock is more sensitive to the changes in moisture content of growth substrate, and could speed up ABA biosynthesis to deliver more ABA to shoots by root pressure than *Cs/Cs* plants. It is highly plausible that such a fast induction of ABA accumulation and associated delivery from the roots to shoots could work as a stress signal and contributed to the higher tolerance against drought for the *Cs/Lc* plants. In agreement with this interpretation, irrigation with AbamineSG, which inhibits the enzyme of 9-cis-epoxycarotenoid dioxygenase (NCED) in ABA biosynthesis^25^, compromised *Lc* rootstock-induced drought tolerance whilst drought tolerance was only partially compromised by foliar application of AbamineSG in the *Cs/Lc* plants (Fig. 5 and Supplementary Fig. S7). Together, these results indicate that luffa rootstock-derived ABA plays a critical role in the enhanced drought tolerance. However, we could not exclude the existence of other signals, such as ACC (ethylene precursor) and cytokinin, from the roots that influence stomatal movement and ABA signaling in the shoots^18^,^42^.

Hypersensitivity to ABA in plants is associated with enhanced tolerance to drought via regulation of stomatal closure and the expression of stress-related genes^43^. In our study, increased sensitivity of stomatal movement to drought in the leaves of *Cs/Lc* plants was associated with increased sensitivity to ABA as evidenced by rapid as well as low concentration-mediated stomatal closure, and the low water loss from the intact leaves (Fig. 6). ABA sensing and signaling are mediated by three classes of proteins: PYR1/PYL/RCAR, PP2C and SnRK2^12^,^26^. In agreement with the increased ABA accumulation and sensitivity, transcript of the ABA receptors genes *PYL1, PYL2, PYL5* and *PYL8,* ABA transporter genes *ABCG22, SnRK2.4* and*SnRK2.5,* which are kinases that interact with PP2Cs, as well as the stress marker genes, *RD29B* and *RAB18* was sharply upregulated whilst the negative regulator gene *PP2C1* was more significantly down-regulated in *Cs/Lc* plants than that in *Cs/Cs* plants in response to water deficit (Fig. 8). The role of these ABA signaling related genes has been well documented^26^,^44^,^45^. For example, overexpression of *PYL5* confers enhanced drought tolerance whilst plants deficient in *ABCG22* are more susceptible to drought stress in Arabidopsis and rice^46^. All these results suggest that the increased sensitivity of stomatal movement to ABA and water deficit is attributable to both the increased ABA accumulation and the induction of the transcript of these ABA signaling genes in *Cs/Lc* plants.

In summary, we demonstrate that the intrinsic drought tolerance ability of rootstock plant (luffa) could greatly influence the behavior of scion plant (cucumber) towards enhanced tolerance to drought. Moreover, rootstock (luffa)-derived ABA serves as a critical signal to regulate both stomatal movement and stomatal development in cucumber. Such developmental alterations, in turn, bring an appropriate balance between CO_2_ assimilation and transpiration, thus increasing WUE and biomass accumulation under drought. In addition to ABA biosynthesis, increased sensitivity of stomata to ABA might play an important role in luffa rootstock-induced drought tolerance. Alterations in the expression of some key genes involved in ABA biosynthesis, perception, signaling and transport have been found to be associated with luffa rootstock-mediated drought tolerance. Such inherent drought tolerance mechanisms could be greatly exploited to improve the agricultural production especially in arid and semi-arid areas.

## Methods

### Plant materials

Two different cucurbit species, cucumber (*Cucumis sativus* L., cv. Jinyan No. 4, *Cs*) and luffa (*Luffa cylindrica* Roem., cv. Xiangfei No. 236, *Lc*), were used as plant materials. Seeds of the two species were sown directly into pots (diameter 15 cm; height 15 cm) filled with a mixture of peat/vermiculite (3/1, v/v). When the cotyledons of the cucumber or luffa (sown for scion) were expanded, a ‘top approach grafting’ was performed^24^. The resulting two groups of seedlings, designated as *Cs/Cs, Cs/Lc, Lc/Cs* and *Lc/Lc* according to the rootstock species cucumber and luffa, respectively, were transferred to growth chambers with the following environmental conditions: 12-h photoperiod, temperature of 25/18 °C (day/night) and photosynthetic photon flux density (PPFD) of 600 μmol m^−2 ^s^−1^. The plants were watered daily and fertilised with Hoagland’s nutrition solution at two days interval. At 4-leaf stage, well-watered plants were used for subsequent water deficit experiments.

### Experiment 1: Reciprocal grafting and WUE experiment

To determine the root-shoot interaction in the drought response, reciprocal grafting between *Cs* and *Lc* was carried out using their scion and rootstock as per following four combinations: *Cs/Cs, Cs/Lc, Lc/Cs* and *Lc/Lc.* Plants at 4-leaf stage were watered to 50% SWC followed by drought imposition through completely withholding water for up to 9 d. In parallel, a group of well-watered plants (50% SWC) were used as control. Water loss was estimated everyday. The total leaf area was measured with a Li 3100 leaf area meter (Li-Cor area meter, model Li 3100). Integrated water-use efficiency (WUE) was determined and calculated as the final shoot dry weight divided by total water loss^5^.

### Experiment 2: Stomatal ABA sensitivity experiment

Stomatal measurements were performed with intact leaves from *Cs/Cs* and *Cs/Lc* plants. Epidermal strips from fully expanded intact leaves were peeled from the abaxial surface with forceps and mesophyll cells were removed from the epidermis using single-edge industrial razor blades^30^. To promote stomatal opening, the epidermis were submerged in stomatal opening buffer (10 mM KCl, 7.5 mM iminodiacetic acid and 10 mM MES, pH 6.2, adjusted with KOH) for 2 h in light (100 μmol m^−2 ^s^−1^) and then incubated for an additional 30 min in stomatal opening buffer supplemented with 0, 10, 20, 50 or 100 μM ABA. Time course of 50 μM ABA-induced changes in stomatal aperture were also observed.

### Experiment 3: ABA biosynthesis inhibition experiment

*Cs/Cs* and *Cs/Lc* plants at 4-leaf stage in the pots were watered to 50% SWC followed by drought imposition through completely withholding water for up to 11 d. At the 2^nd^ and 5^th^ d after the initiation of the drought treatment, shoots were sprayed with 50 μM AbamineSG (5 ml per plant), whereas roots of others were irrigated with 5 μM abamineSG (50 ml per plant) or 10 μM ABA (5 ml per plant), respectively. An equal amount of water was applied as the control.

### Experiment 4: Drought adaptation experiment

Well-watered plants at 4-leaf stage were exposed to five substrate water content (SWC) conditions [50 (well-watered), 40, 30, 15, and 7%] for 7 d. The SWC is expressed as: (volume of water in growth substrate, v)/(volume of growth substrate, v). Water was supplied twice per day to maintain the defined water contents in the growth substrate.

### Leaf gas exchange, chlorophyll fluorescence, water potential and temperature

Gas exchange in fully expanded leaves, including net CO_2_ assimilation (Pn), transpiration (Tr), and stomatal conductance (Gs), was determined using a LI-6400 Portable Photosynthesis System (LI-6400; LI-COR, Lincoln, NE, USA). Gas exchange was measured at a PPFD level of 1000 μmol m^−2 ^s^−1^, a temperature of 25 ^o^C, a relative humidity of 85%, and an ambient CO_2_ concentration of 380 μmol mol^−1^. Instantaneous water-use efficiency was calculated as net CO_2_ assimilation rate divided by transpiration rate. The maximum photochemical efficiency of photosystem II (PSII) (Fv/Fm) was measured using an imaging-PAM chlorophyll fluorimeter equipped with a computer-operated PAM-control unit (IMAG-MAXI; Heinz Walz, Effeltrich, Germany) as previously described^23^. Fv/Fm values were determined using the whole leaf. The leaf water potential (ψ_leaf_) was measured using a Dew point Potential Meter (WP4; Decagon Device, Pullman, USA) on leaves similar to those used for the evaluation of leaf gas exchange. Leaf temperature was imaged by a FLUKE Ti400 ir camera (FLUKE, Washington, USA).

### Water loss from detached leaves

Twelve leaves of similar developmental stage from 12 plants were detached and weighed immediately. They were placed in a temperature controlled growth chamber at 25 ^o^C with 60% humidity and the weight of individual leaves were recorded every hour for 9 h. Water loss from the leaves at each time was calculated as the percentage of the initial fresh weight. The experiment was repeated three times.

### Detection of stomatal aperture, H_2_O_2_ and O_2_^−^ accumulation

The stomatal aperture and stomatal density were measured using a microscope (BX61, Olympus Co., Tokyo, Japan). Hydrogen peroxide production in guard cells were monitored using 2,7-dichlorofluorescein diacetate (H_2_DCF-DA) and the fluorescence of guard cells was observed under a Laser Scanning Confocal Microscopy (Leica TCS SL, Leica Microsystems, Germany) with the excitation wavelength at 480 nm and emission at 530 nm, as previously described^45^. The experiment was repeated three times. The H_2_O_2_ and O_2_^−^ accumulation in the leaves was visualized by 3,3′-diaminobenzidine (DAB) and nitroblue tetrazolium (NBT) staining, respectively^47^.

### Xylem sap collection and ABA measurements

Xylem sap exudates were collected by cutting the stem 5 cm above the junction between the scion and rootstock with a razor blade and then exposing the rootstock to a pressure chamber set to 0.5 MPa. The first two droplets of the xylem sap were discarded to avoid any contamination from injured cells at the cut surface. Then, xylem sap was collected for 20 min using a micro-pipette from the cutting surface into ice-chilled Eppendorf tubes wrapped with aluminium foil. The collected sap samples were frozen immediately in liquid nitrogen and stored at −80 °C until analysis. The extraction and determination of ABA was performed according to the method of Xia *et al*.^30^.

### Antioxidant enzyme activity and relative electrolyte leakage measurements

Frozen leaf segments (0.3 g) were ground with 2 ml ice-cold buffer containing 50 mM PBS (pH 7.8), 0.2 mM EDTA, 2 mM AsA, and 2% (w/v) polyvinylpolypyrrolidone. The homogenates were centrifuged at 4 °C for 20 min at 12,000 *g*, and the resulting supernatants were used for the determination of enzymatic activity. The protein concentration was determined according to Bradford method with bovine serum albumin as standard^48^. The activity of superoxide dismutase (SOD), dehydroascorbate reductase (DHAR) and guaiacol peroxidase (GPOD) were measured following the protocol used as previously described^49^,^50^. Relative electrolyte leakage was estimated as described previously^51^.

### RNA extraction and quantitative real-time PCR (qRT-PCR) analysis

Total RNA was extracted from grafted cucumber leaves using an RNA extraction kit (Axygen, Union City, CA, USA) according to the supplier’s instructions. DNA contamination was removed using a purifying column. One microgram of total RNA was reverse-transcribed using the ReverTra Ace qPCR RT Kit (Toyobo, Osaka, Japan) following the supplier’s recommendations. The gene-specific primers for qRT-PCR were designed based on their cDNA sequences and are listed in Supplemental Table 1. The qRT-PCR assays were performed using an iCycleriQ^TM^ Real-time PCR Detection System (Bio-Rad, Hercules, CA, USA). Each reaction (20 μl) consisted of 10 μl SYBR Green PCR Master Mix (Takara, Tokyo, Japan), 8.6 μl sterile water, 1 μl cDNA, and 0.2 μl forward and reserve primers (10 μM). The PCR conditions consisted of denaturation at 95 °C for 3 min, followed by 40 cycles of denaturation at 95 °C for 30 s, annealing at 58 °C for 30 s and extension at 72 °C for 30 s. The quantification of mRNA levels was based on the method of Livak & Schmittgen^52^.

### Statistical analysis

The experiment was a completely randomised design with four biological replicates. For stomatal aperture analysis, each replicate is the average aperture of 30 randomly selected stomata under each treatment. For others, each replicate contained at least 12 plants. Data were subjected to analysis of variance, and the means were compared using Tukey’s test at the 5% level.

## Additional Information

**How to cite this article**: Liu, S. *et al*. Grafting cucumber onto luffa improves drought tolerance by increasing ABA biosynthesis and sensitivity. *Sci. Rep.* **6**, 20212; doi: 10.1038/srep20212 (2016).

## Supplementary Material

Supplementary Information
