# Supplementary material for: Grafting cucumber onto luffa improves drought tolerance by increasing ABA biosynthesis and sensitivity
Source: Sci Rep. 2016 Feb 2;6:20212. doi: 10.1038/srep20212 (PMC4735794; doi:10.1038/srep20212)
Supplement: Supplementary Information [file srep20212-s1.doc]

**Title:**

**Grafting cucumber onto luffa improves drought tolerance by increasing ABA biosynthesis and sensitivity**

**Authors:**

Shanshan Liu^1^, Hao Li^1,2^, Xiangzhang Lv^1^, Golam Jalal Ahammed^1^, Xiaojian Xia^1^, Jie Zhou^1^, Kai Shi^1^, Tadao Asami^3^, Jingquan Yu^1^, Yanhong Zhou^1,4,*^

*^1^Department of Horticulture, Zijingang Campus, Zhejiang University, 866 Yuhangtang Road, Hangzhou, 310058, P.R. China*

*^2^College of Horticulture, Northwest A&F University, Yangling 712100, P.R. China*

*^3^Department of Applied Biological Chemistry, University of Tokyo, Bunkyo Ku, Tokyo 1138657, Japan*

*^4^Zhejiang Provincial Key Laboratory of Horticultural Plant Integrative Biology, 866 Yuhangtang Road, Hangzhou, 310058, P.R. China*

** To whom correspondence should be addressed. E-mail:* *yanhongzhou@zju.edu.cn*

**Table S1.** Primers used for real time RT-PCR assays.

| Gene | Primer pairs |
| --- | --- |
| *Actin* | F: TGGACTCTGGTGATGGTGTTA |
|  | R: CAATGAGGGATGGCTGGAAAA |
| *NCED2* | F: GTGAACCGAAATCTACTTG |
|  | R: GCTCACCACCATACCTC |
| *PYL1* | F: TTTGGAGATGGACAGGCAGGAG |
|  | R: AAGCATACACCACCATGGACAAAAC |
| *PYL2* | F: GATGCAGGATGCAGTTGAACCAATT |
|  | R: AAACAGAACTCCACACTTCCCCACT |
| *PYL5* | F: CGACAACCCACAGGCTTACA |
|  | R: CGGCATCCCAGTCACCAACA |
| *PYL8* | F: CATTCAAAGGCACCACAACC |
|  | R: GCTCCTGACCAACGACCATA |
| *SnRK2.1* | F: TCGCAACCTTCTTTCTCGC |
|  | R: ATTTCCTCAACGCTCTGTG |
| *SnRK2.2* | F: ATTTGCGACTTCGGCTATTC |
|  | R: TAAGCTCCAACCAGCATCAC |
| *ABCG22* | F: GGAAGGGAATAATACGACAG |
|  | R: TCTGCTCCCAACATAAACAT |
| *PP2C1* | F: TTATGGAGACTGATGCAGCTTTTGC |
|  | R: ATCTCCTATGGCACGTGTAACTCCG |
| *RAB18* | F: GGCACCATCAGCAGCATA |
|  | R: CTTCTTCCTCCTCCCTCC |
| *RD29A* | F: AAGGTGAAGAGCGAAAC |
|  | R: CAGGAGCAAGGCGTTTA |

F indicates forward and R indicates reverse.

**Figure S**1**. Plant growth as influenced by rootstock in reciprocal grafted plants.** Photographs were taken 9 d after water withholding.

**Figure S2**. **Activities of superoxide dismutase (SOD), dehydroascorbate reductase (DHAR) and guaiacol peroxides (GPOD) in leaves as influenced by rootstock and drought stress.** Intact plants were well watered or drought stressed by withholding water for 9 d. Data are presented as the mean of four biological replicates (±SD). Different letters indicate significant differences (*P*<0.05) according to the Tukey’s test.

**Figure S3. Changes in photosynthesis and water use efficiency of luffa plants as influenced by rootstock.** CO_2_ assimilation rate (Pn, a), stomatal conductance (Gs, b), transpiration rate (Tr, c), water loss in growth substrate (d), instantaneous WUE (e) and integrated WUE (f) of luffa grafted plants under well watered and drought stress conditions. Intact plants were well watered (W-*Lc/Lc* and W-*Lc/Cs*) or drought stressed by withholding water (D-*Lc/Lc* and D-*Lc/Cs*) for 9 d. Integrated WUE were determined 9 d after water withholding. Data are presented as the mean of four biological replicates (±SD). Different letters indicate significant differences (*P*<0.05) according to the Tukey’s test.

**Figure S4. Stomatal aperture (a) and H_2_O_2_ accumulation (b) in luffa leaves as influenced by rootstock and drought stress.** Scale bar in (a) = 15 µm. Scale bar in (b) = 50 µm. Intact plants were well watered or drought stressed by withholding water for 9 d. Data are presented as the mean of four biological replicates (±SD). Different letters indicate significant differences (*P*<0.05) according to the Tukey’s test.

**Figure S5. The content of ABA in leaves, roots and xylem in luffa plants in response to rootstock and drought stress**. Intact plants were well watered (W-*Lc/Cs* and W-*Lc/Lc*) or drought stressed (D- *Lc/Cs* and D- *Lc/Lc*) by withholding water for 9 d. Data are presented as the mean of four biological replicates (±SD). Different letters indicate significant differences (*P*<0.05) according to the Tukey’s test.

**Figure S6**. **Stomatal density (a,b) and ABA content (c) in new leaves in response to rootstock and drought stress.** Scale bar = 100 µm. For stomatal density analysis, intact plants were well watered (W-*Cs/Cs* and W-*Cs/Lc*) or drought stressed (D-*Cs/Cs* and D-*Cs/Lc*) by withholding water for 9 d, and then rewatered and allowed to recover for 5 d. ABA content in the new leaves was determined 3 d after drought treatment. Data are presented as the mean of four biological replicates (±SD). Different letters indicate significant differences (*P*<0.05) according to the Tukey’s test.

**Figure S7. Plant growth (a), leaf area per plant (b) and water uptake per plant** **(c)** **as influenced by leaf (L) application or root (R) application of ABA biosynthesis inhibitor or ABA.** Leaf area was determined at 9 d after water withholding. *Cs/Cs* and *Cs/Lc* plants at 4-leaf stage in the pots were watered to 50% SWC followed by drought imposition through completely withholding water for up to 11 d. At the 2^nd^ and 5^th^ d after the initiation of the drought treatment, shoots were sprayed with 50 μM AbamineSG (5 ml per plant), whereas roots of others were irrigated with 5 μM AbamineSG (50 ml per plant) or 10 μM ABA (50 ml per plant). An equal amount of water was applied as the control. Data are presented as the mean of four biological replicates (±SD). Different letters indicate significant differences (*P*<0.05) according to the Tukey’s test.

**Figure S8**. **The leaf temperature and stomatal conductance in response to rootstock and drought stress.** (a, b) Leaf temperature of cucumber plants with its own roots (*Cs/Cs*) and luffa roots (*Cs/Lc*) as rootstocks under different substrate water content conditions. (c) Stomatal conductance (Gs) as influenced by rootstocks and substrate water contents. All data were determined 7 d after drought treatment. Data are presented as the mean of four biological replicates (±SD). Different letters indicate significant differences (*P*<0.05) according to the Tukey’s test.
